# Controllable intein splicing and N-terminal cleavage at mesophilic temperatures

**DOI:** 10.3389/fbioe.2025.1543573

**Published:** 2025-02-07

**Authors:** Taylor A. McNeal, Joel Weinberger, Geraldy L. S. Liman, Tia M. Ariagno, David W. Wood, Thomas J. Santangelo, Christopher W. Lennon

**Affiliations:** ^1^ Department of Biological Sciences, Murray State University, Murray, KY, United States; ^2^ Department of Biochemistry and Molecular Biology, Colorado State University, Fort Collins, CO, United States; ^3^ William G. Lowrie Department of Chemical and Biomolecular Engineering, The Ohio State University, Columbus, OH, United States

**Keywords:** intein, protein splicing, N-terminal cleavage, bioseparations, biosensor

## Abstract

Inteins (intervening proteins) interrupt host proteins and are removed through a protein splicing reaction that ligates adjacent N- and C-exteins. The ability of inteins to specifically rearrange peptide bonds has proven exceptionally useful in protein engineering, thus, methods to control intein activity are of considerable interest. One particularly useful application of inteins is for the removal of an affinity tag following purification of a target protein through N-terminal cleavage (NTC). Typically, extended incubation at high temperature (greater than 50°C) or with an external nucleophile (e.g., dithiothreitol) is required to drive NTC, conditions that compromise the folding of many target proteins. Here, we characterize a variant of the *Thermococcus kodakarensis* RadA intein that can perform NTC at moderate temperatures in the absence of an external nucleophile. While we find that while NTC is largely inhibited during expression in *Escherichia coli* at 15°C, rapid and efficient NTC can be activated 37°C. Our results provide an alternative intein-based system – one that does not require either an external nucleophile or prolonged incubation at high temperature to stimulate NTC – that controls intein activity within a temperature range amenable to most mesophilic experimental organisms.

## 1 Introduction

**FIGURE 1:**
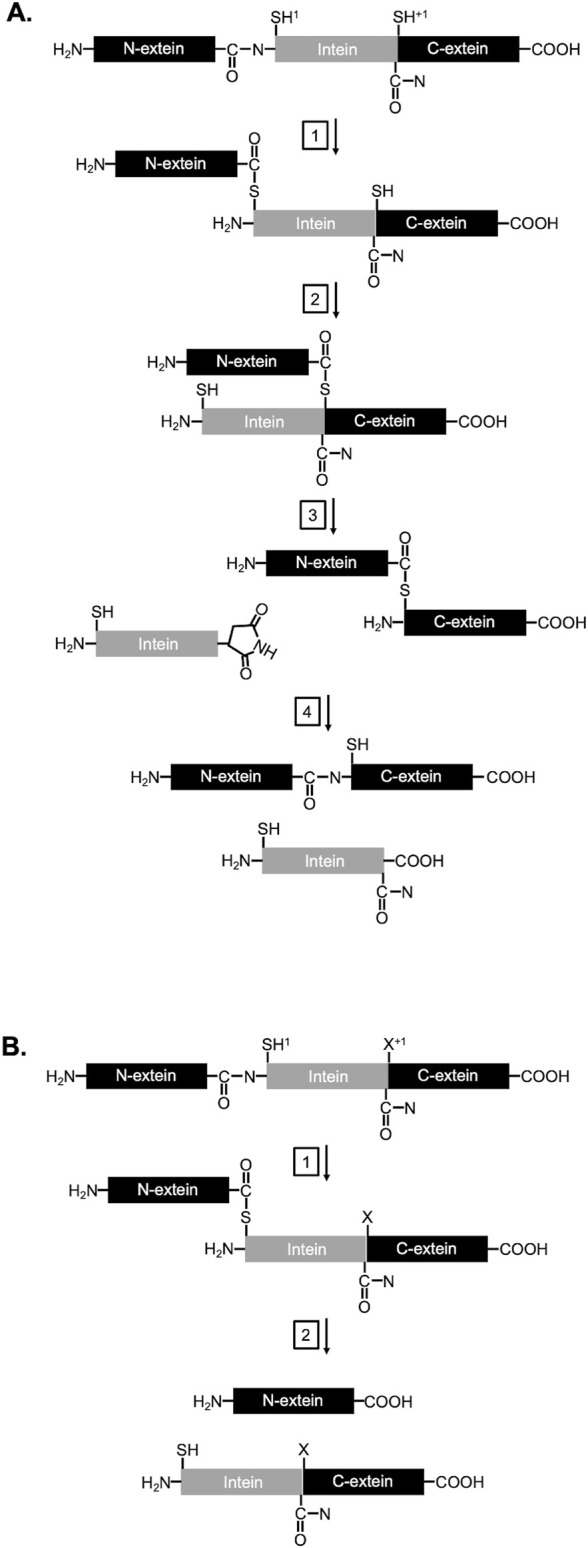
Mechanism of protein splicing and N-terminal cleavage: **(A)** Splicing and **(B)** NTC schemes with −1, 1, and +1 positions, exteins (black), and intein (gray) indicated. Relevant chemical groups essential to protein splicing and NTC are drawn. Details for each step are described in the main text.

Inteins, or intervening proteins, are mobile genetic elements removed from host proteins through protein splicing. In this process, the intein removes itself by rearranging two peptide bonds and rejoining the flanking sequences, termed N-and C-exteins, with a new peptide bond. In the canonical (class 1) mechanism of protein splicing, the reaction proceeds in four steps (Figure 1A; reviewed in Mills et al. (2014), Wood et al. (2023). First, a nucleophilic attack by the first residue of the intein (cysteine or serine) on the preceding peptide bond forms a linear (thio) ester. Second, a cysteine, serine, or threonine at the first position of the C-extein performs a second nucleophilic attack on the (thio) ester from step 1, forming a branched intermediate. Third, the last residue of the intein, an asparagine, cyclizes to release the intein. Fourth, the (thio) ester linking the N- and C-exteins rearranges to form a peptide bond, generating the uninterrupted host protein (ligated exteins).

The ability of inteins to rearrange peptide bonds in a specific and controlled manner has been exploited by engineers to develop numerous technologies, including platforms for protein purification, segmental isotope labeling, formation of cyclic peptides, incorporation of non-natural modules into proteins, fabrication of protein arrays, sensor development, imaging, and regulation of protein function *in vivo* [reviewed in Wood and Camarero (2014), Sarmiento and Camarero (2019), Wood et al. (2023)]. While accurate protein splicing typically dominates for most inteins, off-pathway reactions are possible. For example, N-terminal cleavage (NTC) can occur when the (thio)ester formed between the N-extein and intein is cleaved by an external nucleophile prior to ligation to the C-extein (Figure 1B) (Mills et al., 2014). Several characterized mutations near the intein/extein junctions, as well as solution condition, can favor these cleavage reactions (Mills et al., 2014). Inteins with cleavage, but not splicing activities, have been particularly valuable for protein purification strategies. In one widely used intein-based purification technology (Wood et al., 1999; Prabhala et al., 2022), a target protein serves as one extein while an affinity tag serves as the other extein. Modified intein activity – resulting from mutations that block splicing but accelerate N-terminal cleavage – releases the purified target from the affinity tag at the end of the affinity purification process (Supplementary Figure S1). Thus, intein-based protein purification systems provide convenient and cheaper affinity tag/intein removal from the final target protein without the need for costly exogenous proteases.

Although intein C-terminal cleaving is spontaneous and can be made pH or temperature sensitive, conventional NTC systems require either extended incubation with high concentrations of an external nucleophile (e.g., Dithiothreitol; DTT) or high temperature (greater than 50°C) (Mills et al., 2006; Amitai et al., 2009). While these systems have proven exceptionally useful, the conditions necessary to drive efficient cleavage (e.g., large temperature increases) often disrupt the folding of target proteins. Furthermore, the addition of an external nucleophile (e.g., DTT) at high concentrations can be prohibitively expensive for large-scale applications and is expected to disrupt disulfide bonds in proteins that possess them. Here, we report a variant of the *Thermococcus kodakarensis* RadA intein that performs controllable protein splicing and NTC at modest temperatures and that is efficient in the absence of DTT. This intein greatly expands the use of intein-based NTC to target proteins whose activity is sensitive to reducing agents and high temperatures.

## 2 Materials and methods

### 2.1 Plasmids

**FIGURE 2:**
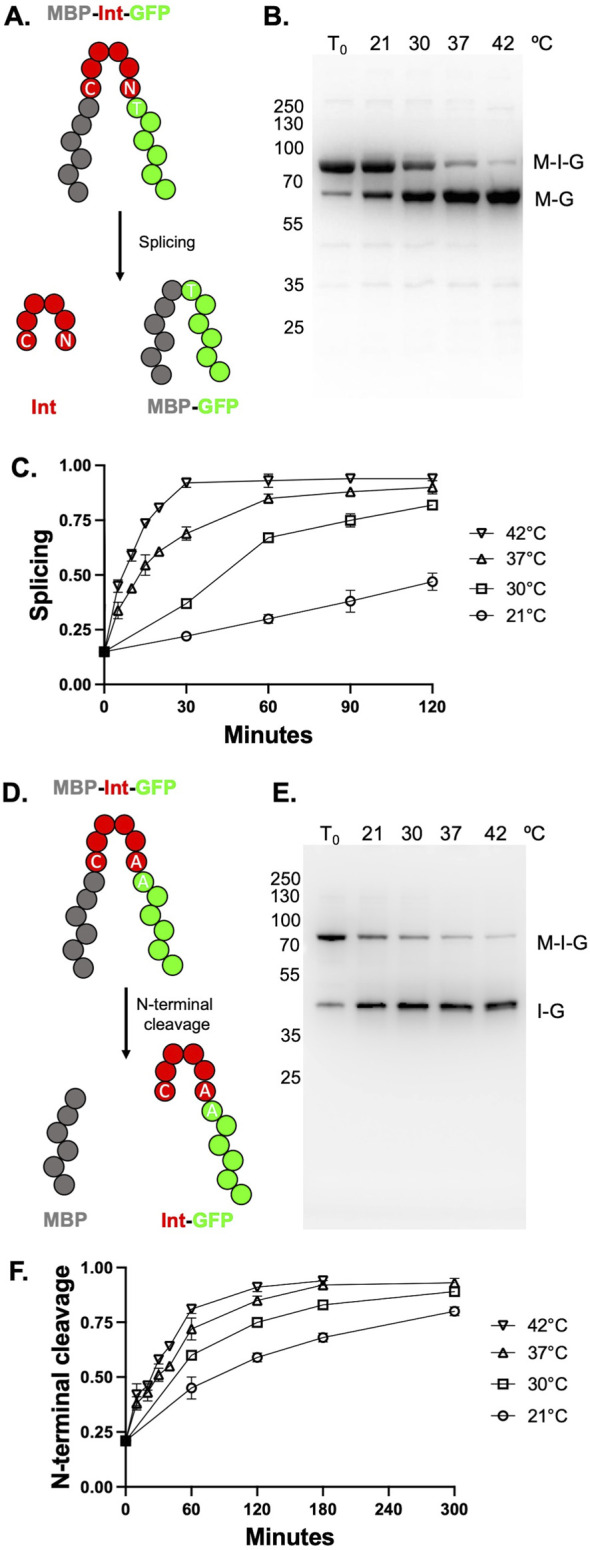
TkΔE splicing and TkΔE-AA N-terminal cleavage (NTC) is activated by increasing temperature. **(A)** Schematic of MBP-Intein-GFP (MIG) reporter. MBP is colored gray, intein is colored red, and GFP is colored green. GFP-containing fluorescent products are visualized in-gel following semi-native PAGE. **(B)** Splicing of TkΔE in MIG reporter at different temperatures for 60 min. Precursor (M-I-G) and ligated exteins (M-G) bands are indicated. **(C)** Quantification of splicing at different temperatures from three independent splicing reactions. **(D)** MIG reporter with mutations to allow for NTC rather than splicing. Colored as in panel **(A) (E)** NTC TkΔE-AA in MIG reporter over 180 min at different temperatures. Precursor (M-I-G) and intein-GFP (I-G) products are indicated. **(F)** Quantification of splicing at different temperatures from three independent cleavage reactions. Error bars indicate standard deviation. When error bars are not shown in panels C and F, they are smaller than the symbol. Protein size markers are in kilodaltons.

All reporter constructs described in this manuscript were mutagenized, sequenced, and prepared commercially (Genscript) using the previously described MIG genefusion-containing plasmid (Weinberger II and Lennon, 2021), which confers chloramphenicol resistance and MIG gene expression under control of a T7-inducible promoter. In this MIG reporter, an intein (with 10 N-terminal and 10 C-terminal residues from the native RadA exteins) is flanked by the *Escherichia coli* maltose binding protein (MBP) with an N-terminal His-tag (His-MBP) as the N-extein and the superfolder green fluorescent protein (GFP) as the C-extein (Figure 2A) (Topilina et al., 2015).

### 2.2 Protein expression

Following plasmid transformation into *E. coli* BL21(DE3) (New England Biolabs), cells were grown with shaking at 250 rpm in LB broth with 25 μg/mL chloramphenicol at 37°C to an optical density of approximately 0.5 at 600 nm, culture temperature was reduced to 15°C with shaking at 250 rpm continued, and protein expression was induced by addition of 1 mM isopropyl β-D-1-thiogalactopyrandoside. After approximately 20 h of protein expression at 15°C, cultures were centrifuged at 4,000×*g* to harvest cells.

### 2.3 Splicing and N-terminal cleavage assays

Following protein expression, cell pellets were suspended in 1 × PBS (11.9 mM phosphates, 137 mM sodium chloride, 2.7 mM potassium chloride, pH 7.4), lysed by sonication, and insoluble material was removed by centrifugation at 16,100×*g*. Whole cell supernatant (WCS) containing the expressed MIG protein was used to monitor splicing or NTC. For time zero (T_0_), WCS was mixed with Bio-Rad 4× Laemmli sample buffer following centrifugation and stored at −20°C. To purify the MIG protein referred to as TkPl-AA, a construct where the HEN adjacent residues of Tk RadA intein were mutated to match those of the HEN-lacking *Pyrococcus horikoshii* RadA mini-intein (described further in Results) for NTC analyses, cell pellets were resuspended in 20 mM Tris (pH 8.0), 30 mM imidazole, and 500 mM sodium chloride. Following lysis by sonication, MIG TkPl-AA was isolated via an N-terminal His-tag from WCS using Ni-Charged MagBeads (Genscript). Purified protein was eluted in 20 mM Tris (pH 8.0), 500 mM imidazole, and 500 mM sodium chloride, then dialyzed into 1X PBS prior to NTC assays. For all splicing and NTC assays, including on-column cleavage, samples were incubated at the temperature and time indicated in Results and Figures. To stop reactions, samples were mixed with Bio-Rad 4× Laemmli sample buffer and stored at −20°C. Where applicable, ZnCl_2_ was present at 10 mM and external nucleophiles (DTT and HA) were present at 50 mM.

### 2.4 SDS-PAGE

In all assays except Figure 4C, in-gel fluorescence is used to visualize GFP-containing fluorescent products following semi-native PAGE as described (Weinberger II and Lennon, 2021). Briefly, reaction mixtures were separated using Tris-glycine TGX gels (Bio-Rad) where samples were not heated prior to separation in order to maintain GFP structure. Coomassie staining was used to visualize all products in Figure 4C.

### 2.5 Imaging and data analysis

GFP-containing products were observed using in-gel fluorescence measurements immediately following SDS-PAGE by an Amersham Imager 680 (GE Healthcare). Coomassie-stained products were visualized using white light. In all cases, ImageJ (https://imagej.nih.gov/ij/) was used to measure relative band amounts by densitometry. Average NTC or splicing, as well as standard deviation, was calculated at each time point by averaging three independent reactions and plotted using Prism (GraphPad).

## 3 Results

### 3.1 Conditional protein splicing is possible at mesophilic temperatures

We identified and characterized a variant of the intein naturally located within the *T. kodakarensis* RadA protein that displayed unique activity when placed within a splicing reporter construct and expressed within *E. coli*. Deletion of the homing endonuclease domain (residues 276-585) from the native intein generated a variant (referred to as TkΔE) that was defective for splicing within our reporter construct when expressed at low temperature (15°C), but that spliced efficiently at moderately high temperatures (50°C) (Liman et al., 2024). Given the potential to exploit TkΔE for protein purification, we sought to probe the temperature-dependent rescue of TkΔE splicing in more detail, reasoning that, if splicing could be activated at modest temperatures, then TkΔE could be useful for intein applications at temperatures that are physiologically relevant for many model organisms.

We measured splicing and cleavage efficiencies using a MIG reporter containing the TKΔE intein (see Materials and methods). Precursor and splicing or cleavage product ratios can be monitored by in-gel fluorescence following semi-native PAGE (Weinberger II and Lennon, 2021). Note that MIG assays are performed within crude cell supernatants unless otherwise described. Following expression for 20 h in *E. coli* at 15°C (T_0_), we observe that very little splicing had occurred *in vivo*, with approximately 85% fluorescent products as unspliced precursor (Figures 2B, C). In contrast to poor splicing efficiencies observed *in vivo* at 15°C during protein expression and cell lysis, incubation of the TkΔE intein in the MIG reporter at 37°C and 42°C resulted in increased and then near total splicing within 1 hour. At 30°C, approximately half of the precursor had spliced after 1 hour (Figures 2B, C). Splicing was possible but substantially slower at 21°C, with approximately 25% of the precursor converting to ligated exteins after 2 hours (Figures 2B, C).

### 3.2 TkΔE permits conditional NTC at mesophilic temperatures

NTC is particularly useful for intein-mediated affinity tag removal from a protein of interest following purification. However, conditional splicing by an intein does not necessarily mean that NTC will proceed under similar conditions, particularly without an external nucleophile. Therefore, we next wanted to examine if TkΔE could undergo NTC in response to temperature in a manner similar to splicing. To promote NTC instead of splicing, the last residue of the intein, a conserved Asn, and the first residue of the C-extein, a conserved Thr, were changed to Ala (TkΔE-AA; Figure 2D). After expression at 15°C in *E. coli* for 20 h, only approximately 25% of the precursor had undergone NTC to form His-MBP and intein-GFP (Figure 2E). Incubation of TkΔE-AA in crude lysates lacking an external nucleophile at temperatures ranging from 21°C to 42°C stimulated NTC. As with TkΔE splicing, higher temperatures led to an increasing rates of NTC for TkΔE-AA (Figures 2E, F).

### 3.3 TkΔE protein splicing and TkΔE-AA NTC is inhibited by zinc

Zinc is known to be a potent inhibitor of intein-mediated protein splicing; however, this inhibition can be reversed by introducing a chelator, such as ethylenediaminetetraacetic acid (EDTA) (Mills and Paulus, 2001; Woods et al., 2020). Inhibition can be mediated through direct binding of zinc to the initiating nucleophilic residue of the intein required for splicing and NTC (Woods et al., 2020). We therefore probed whether zinc addition could diminish or inhibit the splicing and NTC actitivites of TkΔE and TkΔE-AA, respectively. As expected, the addition of just 10 mM zinc is sufficient to radically diminish both splicing and NTC (Supplementary Figures S2A–C), providing a second mechanism (with termperature control as the first) to control conditional protein splicing and NTC activities of the TkΔE intein.

### 3.4 The TkΔE intein can efficiently drive NTC without an external nucleophile

For existing intein-based protein purification schemes utilizing NTC as a means of affinity tag removal, efficient NTC can be driven at lower temperatures by the addition of high concentrations of an external nucleophile. One of the most commonly used is DTT, which promotes thiolysis of the scissle thioester bond between the N-extein and intein (Prabhala et al., 2022). However, DTT reduces disulfide bonds that may be required for proper folding of some proteins and the required concentrations would be prohibitively expensive for large-scale preparations, limiting the value of driving NTC through DTT addition. Therefore, an intein capable of NTC in the absence of DTT could be useful for certain applications. Excitingly, TkΔE-AA can efficiently undergo NTC in the absence of an external nucleophile (e.g., DTT) (Figures 2E, F) at mesophilic temperatures. We compared the rates of NTC in the presence and absence of DTT and, surprisingly, found that the rate of cleavage is not accelerated by DTT (Supplementary Figures S3A, B). Interestingly, the smaller external nucleophile hydroxylamine (HA), which is also known to accelerate the rate of NTC (Amitai et al., 2009), increases the cleavage rate by approximately 5-fold (Supplementary Figures S3A, B). Therefore, the smaller external nucleophile HA, but not the larger DTT, is capable of accelerating NTC. Of note, HA can lead to side reactions (Asn-Gly bond cleavage) and human toxicity, which may limit the use of this nucleophile for large-scale applications.

### 3.5 HEN domain deletion does not inhibit NTC

Given our observation that DTT does not stimulate TkΔE-AA NTC, we were interested in whether this was an inherent property of the *T. kodakarensis* RadA intein or due to deletion of the HEN domain. We previously found that splicing was robust for the *T. kodakarensis* RadA intein with the HEN domain present, with greater than 90% splicing during expression in *Escherichia coli* at 15°C (Liman et al., 2024). Note that the HEN domain’s active site (residues 373-381) were mutated to prevent DNA cleavage and toxicity to *E. coli*. To determine whether TkE could perform NTC, we mutated the terminal Asn of the intein and +1 residue of the C-extein, referred to here as TkE-AA, which is capable of NTC but not splicing. This variant is identical to TkΔE-AA, except for the presence of the HEN domain. Following overnight expression in *E. coli* at 15°C, we find that approximately 90% of TkΔE-AA has undergone NTC, compared to just 25% for TkΔE-AA (Supplementary Figure S3C). These results are consistent with previous observations that the *T. kodakarensis* RadA intein activity increases when the HEN domain is present (Liman et al., 2024), likely by stabilizing the intein fold and/or promoting formation of the intein active site.

### 3.6 Each amino acid at the −1 position supports controlled and efficient NTC

**FIGURE 3:**
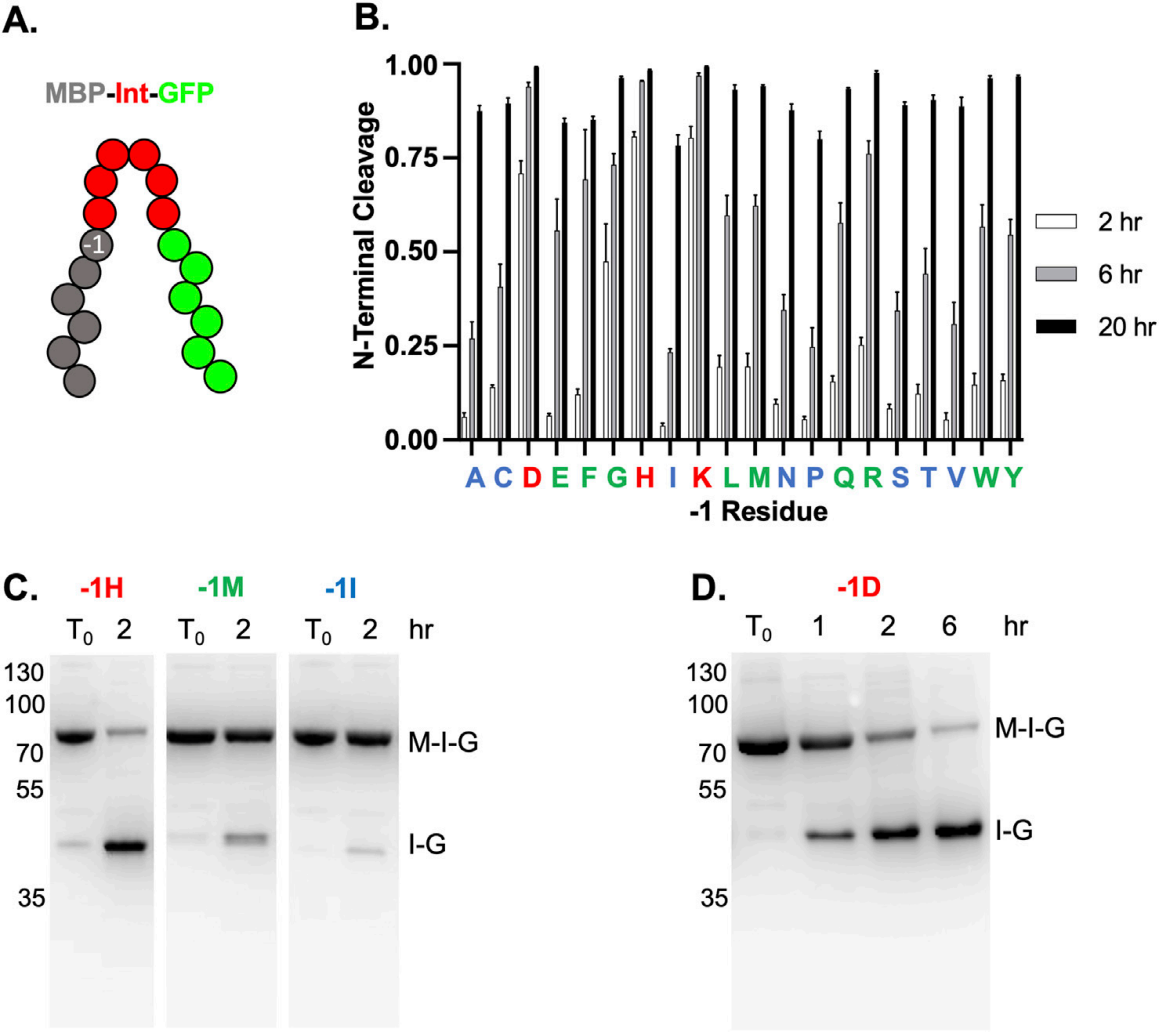
TkΔE-AA NTC with 20 common amino acids in the -1 position. **(A)** Schematic of MBP-Intein-GFP (MIG) reporter with the −1 position indicated. Exteins and intein colored as in Figure 1. **(B)** Quantification of NTC with different residues at −1 position of TkΔE-AA after incubation for 2 (white bars), 6 (gray bars), and 20 (black bars) hours at 37°C. The relative rates of NTC for each −1 position, as described in the main text, are indicated with red (fast), green (moderate), or blue (slow). One letter amino acids abbreviations are shown. **(C)** Examples of relative NTC rates for a fast, moderate, and slow −1 residue. NTC after 2 h at 37°C for TkΔE-AA with -1H (fast), -1M (moderate), and -1I (slow). **(D)** TkΔE-AA-1D (Asp in −1 position) does not undergo premature NTC following expression at 15°C for 20 h and proceeds following incubation at 37°C for indicated times. Quantification in panel A is done as described in Figure 1. Protein size markers are in kilodaltons.

The identity of the last residue of the N-extein, known as the −1 position, has been shown to drastically influence the rate of NTC (Amitai et al., 2009) (Figure 3A. We, therefore, investigated how the identity of the −1 residue affected NTC by TkΔE-AA in the MIG context, examining all twenty amino acids in the −1 position after a 2, 6, or 20-hour incubation at 37°C. As expected, the rate of NTC was highly variable depending on the identity of the residue in the −1 position, with the native −1 residue for TkΔE-AA, lysine, displaying the fastest NTC rate (Figure 3B). We classified the twenty −1 TkΔE-AA residue variants into fast, moderate, or slow based on NTC rate, with fast demonstrating greater than 50% NTC after 2 h, moderate greater than 50% NTC after 6 h, and slow less than 50% NTC after 6 h (Figure 3B). In addition to lysine, only histidine and aspartate fell into the fast category, with nine residues displaying a relatively moderate rate of NTC (glutamate, phenylalanine, glycine, leucine, methionine, glutamine, arginine, tryptophan, tyrosine), and the remaining eight residues undergo NTC at a relatively slow rate (alanine, cysteine, isoleucine, asparagine, proline, serine, threonine, valine) (Figure 3B). An example from each rate category following incubation at 37°C for 2 h is shown (Figure 3C). A clear pattern of NTC rate based on the physical nature of the −1 position side chain was not readily apparent. Importantly, while the NTC rate is highly variable depending on the −1 position, all residues display nearly complete NTC following a 20-h incubation at 37°C, although not all target proteins will be amenable to these conditions.

### 3.7 TkΔE-AA permits controlled NTC with aspartate at the −1 position

For many inteins, an aspartate at the −1 position leads to premature NTC, even if the construct is capable of productive splicing (Oeemig et al., 2012; Amitai et al., 2009). For example, the *Pyrococcus horikoshii* (Ph) RadA intein primarily undergoes NTC, rather than splicing, with aspartate as the −1 residue, even without mutations to favor NTC (Oeemig et al., 2012). This effectively excludes the use of intein-based purification strategies to proteins that do not end in aspartate. Interestingly, we demonstrate that for TkΔE-AA, aspartate in the −1 position does not lead to premature cleavage (Figure 3D). Following expression in *E. coli* for 20 h at 15°C, less than 5% NTC has occurred (Figure 3D). After incubation at 37°C, NTC proceeds rapidly, with nearly 75% NTC after only 2 h (Figure 3D).

### 3.8 TkΔE-AA can be minimized without compromising functionality

For TkΔE-AA to be more useful as a potential means for affinity tag removal, the number of residues dividing the N-extein from the intein should be minimized. The MIG reporter has a spacer between MBP and the intein that includes a Factor Xa cleavage site and ten residues from the native N-extein. To investigate whether these sequences could be removed without compromising NTC, we eliminated these residues, fusing the His-MBP tag with a single lysine residue on the C-terminus to TkΔE-AA. To compensate for any potential loss in intein activity, we changed the residues surrounding the homing endonuclease insertion site of the Tk RadA intein to match those of the Ph RadA intein, a mini-intein that naturally lacks a homing endonuclease. We previously found that these substitutions increase TkΔE splicing (Liman et al., 2024). We refer to this intein as TkPl-AA. A comparison of the TkΔE and Ph RadA intein residues is provided (Supplementary Figure S4).

**FIGURE 4:**
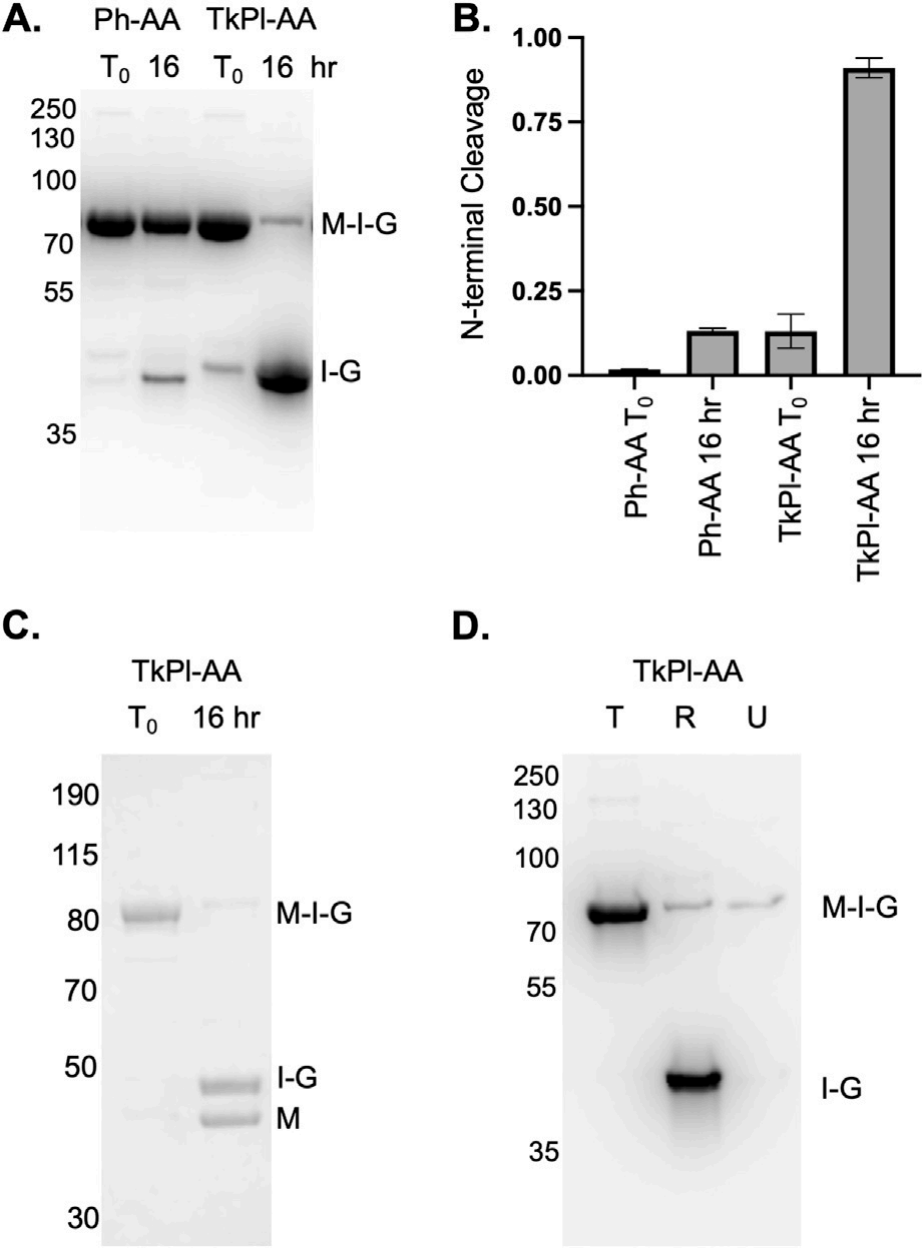
Homologous intein from *Pyrococcus horikoshii* is not prone to NTC and on-column NTC of the TkPl-AA variant. **(A)** TkPl-AA, but not Ph-AA, in the MIG reporter undergoes efficient NTC during incubation at 37°C for 16 h. **(B)** Quantification of NTC for TkPl-AA and Ph-AA following incubation at 37°C for 16 h **(C)** TkPl-AA MIG precursor intein can be purified, and, after incubation for 16 h at 37°C, two products are observed by Coomassie staining with sizes consistent with the cleaved MBP and intein-GFP. **(D)** TkP1-AA MIG precursor intein maintains NTC activity at 37°C while on beads following affinity resin capture and washing. In panel D, T indicates the product eluted from total amount of beads prior to incubation at 37°C, R is the product released into solution following incubation at 37°C, and U is the product that remained on the beads following incubation at 37°C. In all panels, T_0_ is as described in Figure 1 and precursor (M-I-G), MBP (M) and intein-GFP (I-G) bands are indicated. Quantification and error are determined based on at least three independent NTC reactions as described in Figure 1. Protein size markers are in kilodaltons.

Following TkPl-AA expression at 15°C for 20 h, minimal NTC is observed (Figures 4A, B), yet, upon incubation of TkPl-AA in the absence of an external nucleophile at 37°C for 16 h, we find greater than 90% NTC has occurred (Figures 4A, B). We wondered if the ability to perform NTC under these conditions was unusual compared to a similar intein. To answer this question. we compared NTC rates of TkPl-AA to the RadA intein from *P. horikoshii*. Both the Ph and TkPl RadA inteins are the same length and are greater than 75% identical in residue sequence. When incubating the Ph RadA intein, with the terminal asparagine of the intein and +1 threonine mutated to alanine, and in an identical extein context (Ph-AA), we observe minimal NTC compared to TkPl-AA (Figures 4A, B). Interestingly, while Ph-AA NTC is inhibited compared to TkPl-AA, we previously found that splicing of the Ph RadA intein is more efficient than the TkPl intein (Liman et al., 2024).

### 3.9 N-terminal cleavage of purified TkPl-AA

We sought to evaluate TkPl-AA in a real-world context as a potential tool for affinity tag removal, a common application for inteins, but one that currently requires high concentrations of DTT or extended incubation above 50°C. Following purification using immobilized metal affinity chromatography, the TkPl-AA MIG precursor (His-MBP-intein-GFP) can be isolated with minimal NTC (Figure 4C), which is ideal as premature cleavage reduces purification yields. While the elimination of the spacer between MBP and the intein slows the rate of NTC for TkPl-AA compared to TkΔE-AA, this largely blocks premature NTC prior to and during purification (T_0_; Figure 4C). Following incubation at 37°C for 16 h at neutral pH in the absence of an external nucleophile, virtually all precursor underwent NTC, resulting in two bands corresponding in size to His-MBP (42.6 kDa) and TkPl-AA-GFP (47.8 kDa). These bands are both visible following Coomassie staining (Figure 4C).

During purification, the TkP1-AA MIG precursor is bound to metal affinity chromatography beads via an N-terminal His-tag (Figure 4D). From this point, the precursor can either be eluted, or can remain bound to the beads and incubated at 37°C to induce NTC. When TkP1-AA precursor is bound to the beads and incubated at 37°C for 24 h, a majority of the precursor remains active, undergoing NTC (Figure 4D). This results in release of intein-GFP into solution during the incubation, whereas His-MBP remains bound to the beads. This provides a useful strategy for separating a target protein (e.g., His-MBP) from unwanted components (e.g., intein-GFP) directly on affinity beads (Figure 4D).

## 4 Discussion

Conditional protein splicing, wherein the rate and accuracy of protein splicing is dependent on an external signal, naturally occurs for several inteins and has even been engineered through directed evolution (Buskirk et al., 2004; Skretas and Wood, 2005; Lennon and Belfort, 2017; Wood et al., 2023). While temperature has previously been used as an effective signal to stimulate splicing and NTC (Mills et al., 2006; Mills et al, 2004), here we report variants of the *T. kodakarensis* RadA intein with controllable splicing (TkΔE) and NTC (TkΔE-AA and TkPl-AA) activity at lower temperatures. For all variants, we find that intein activity is largely blocked during expression at 15°C in *E. coli*, but that the intein can efficiently undergo splicing and NTC at temperatures greater than 20°C. In another intein-based, temperature-dependent system for NTC, incubation for 5 h at 55°C was needed to stimulate approximately 70% NTC (Mills et al., 2006). It is of note that this previous system did not require the addition of an external nucleophile (e.g., DTT). This is comparable to the level of NTC from TkΔE-AA incubated for the same amount of time at just 21°C. Therefore, compared to this previously reported system, NTC by TkΔE-AA occurs at a substantially lower temperature.

We demonstrate that TkΔE-AA and TkPl-AA variants do not require an external nucleophile to stimulate NTC. Interestingly, we find that while DTT does not accelerate TkΔE-AA NTC, the smaller nucleophile HA increases the rate of NTC. These results suggest the scissile bond between the N-extein and intein is in an unusual conformation compared to other inteins as it is accessible to HA, but not to the larger DTT.

TkΔE-AA can accommodate all 20 common amino acids in the −1 position, with the rate of NTC varying depending on residue identity. Importantly, all undergo near complete NTC after incubation at 37°C for 20 h. This suggests that this intein variant could accommodate any C-terminal residue of a protein of interest (POI) to be purified. Interestingly, we observe that aspartate can be accommodated in the −1 position of TkΔE-AA without premature NTC. This appears to solve the limitation of previous intein-based systems, expanding potential intein uses for proteins with a C-terminal aspartate.

We show that the RadA mini-intein from *P. horikoshii*, which is homologous to the *T. kodakarensis* RadA intein except that it naturally lacks a HEN domain (Supplementary Figure S4), is not prone to NTC under conditions where TkPl-AA cleavage is efficient. Finally, we demonstrate the potential of the TkPl-AA intein, a fusion between TkΔE-AA and the Ph intein, to efficiently undergo NTC while bound to affinity resin at 37°C. These results suggest that the TkPl-AA intein may be useful for intein-based purification systems at moderate temperatures in the absence of an external nucleophile. In this proposed purification scheme, the TkPl-AA intein and affinity tag (AT) would be fused to the C-terminus of a POI to be purified. Following overexpression at 15°C, the POI-intein-AT precursor protein would be bound to chromatography resin via the AT at temperatures below 20°C. After washing away impurities, TkPl-AA-mediated NTC would be induced by temperature at or above 37°C, resulting in the elution of the pure, tagless POI (Supplementary Figure S1). For convenience in this work, we decided to evaluate NTC activity using our MIG reporter, which has the affinity tag fused to the N-terminus rather than the C-terminus. To purify a tagless protein of POI through NTC, the affinity tag should be fused to the C-terminus of the fusion protein.

To our knowledge, our intein variants (TkΔE, TkΔE-AA, and TkPl-AA) display a unique range of temperature-activated splicing and NTC. This relatively fast splicing and NTC at 37°C and comparatively low activity during expression at 15°C represents both a temperature range and time scale that could prove useful for many organisms of interest to the research community. As a whole, these results represent useful discoveries that expand the potential application of intein-based systems.

## Data Availability

The original contributions presented in the study are included in the article/Supplementary Material, further inquiries can be directed to the corresponding author.

## References

[B1] Amitai G. Callahan B. P. Stanger M. J. Belfort G. Belfort M. (2009). Modulation of intein activity by its neighboring extein substrates. Proc. Natl. Acad. Sci. U. S. A. 106, 11005–11010. 10.1073/pnas.0904366106 19541659 PMC2708771

[B2] Buskirk A. R. Ong Y.-C. Gartner Z. J. Liu D. R. (2004). Directed evolution of ligand dependence: small-molecule-activated protein splicing. Proc. Natl. Acad. Sci. U. S. A. 101, 10505–10510. 10.1073/pnas.0402762101 15247421 PMC489967

[B3] Lennon C. W. Belfort M. (2017). Inteins. Curr. Biol. 27, R204–R206. 10.1016/j.cub.2017.01.016 28324730

[B4] Liman G. L. S. Lennon C. W. Mandley J. L. Galyon A. M. Zatopek K. M. Gardner A. F. (2024). Intein splicing efficiency and RadA levels can control the mode of archaeal DNA replication. Sci. Adv. 10, eadp4995. 10.1126/sciadv.adp4995 39292776 PMC11409957

[B5] Mills K. V. Connor K. R. Dorval D. M. Lewandowski K. T. (2006). Protein purification via temperature-dependent, intein-mediated cleavage from an immobilized metal affinity resin. Anal. Biochem. 356, 86–93. 10.1016/j.ab.2006.04.055 16756933

[B6] Mills K. V. Johnson M. A. Perler F. B. (2014). Protein splicing: how inteins escape from precursor proteins. J. Biol. Chem. 289, 14498–14505. 10.1074/jbc.R113.540310 24695729 PMC4031507

[B7] Mills K. V. Manning J. S. Garcia A. M. Wuerdeman L. A. (2004). Protein splicing of a Pyrococcus abyssi intein with a C-terminal glutamine. J. Biol. Chem. 279, 20685–20691. 10.1074/jbc.M400887200 15024006

[B8] Mills K. V. Paulus H. (2001). Reversible inhibition of protein splicing by zinc ion. J. Biol. Chem. 276, 10832–10838. 10.1074/jbc.M011149200 11152694

[B9] Oeemig J. S. Zhou D. Kajander T. Wlodawer A. Iwaï H. (2012). NMR and crystal structures of the Pyrococcus horikoshii RadA intein guide a strategy for engineering a highly efficient and promiscuous intein. J. Mol. Biol. 421, 85–99. 10.1016/j.jmb.2012.04.029 22560994 PMC3392434

[B10] Prabhala S. V. Gierach I. Wood D. W. (2022). The evolution of intein-based affinity methods as reflected in 30 years of patent history. Front. Mol. Biosci. 9, 857566. 10.3389/fmolb.2022.857566 35463948 PMC9033041

[B11] Sarmiento C. Camarero J. A. (2019). Biotechnological applications of protein splicing. Curr. Protein Pept. Sci. 20, 408–424. 10.2174/1389203720666190208110416 30734675 PMC7135711

[B12] Skretas G. Wood D. W. (2005). Regulation of protein activity with small-molecule-controlled inteins. Protein Sci. 14, 523–532. 10.1110/ps.04996905 15632292 PMC2386410

[B13] Topilina N. I. Green C. M. Jayachandran P. Kelley D. S. Stanger M. J. Piazza C. L. (2015). SufB intein of *Mycobacterium tuberculosis* as a sensor for oxidative and nitrosative stresses. Proc. Natl. Acad. Sci. U. S. A. 112, 10348–10353. 10.1073/pnas.1512777112 26240361 PMC4547236

[B14] Weinberger II J. Lennon C. W. (2021). Monitoring protein splicing using in-gel fluorescence immediately following SDS-PAGE. Bio Protoc. 11, e4121. 10.21769/BioProtoc.4121 PMC8413630 34541040

[B15] Wood D. W. Belfort M. Lennon C. W. (2023). Inteins-mechanism of protein splicing, emerging regulatory roles, and applications in protein engineering. Front. Microbiol. 14, 1305848. 10.3389/fmicb.2023.1305848 38029209 PMC10663303

[B16] Wood D. W. Camarero J. A. (2014). Intein applications: from protein purification and labeling to metabolic control methods. J. Biol. Chem. 289, 14512–14519. 10.1074/jbc.R114.552653 24700459 PMC4031509

[B17] Wood D. W. Wu W. Belfort G. Derbyshire V. Belfort M. (1999). A genetic system yields self-cleaving inteins for bioseparations. Nat. Biotechnol. 17, 889–892. 10.1038/12879 10471931

[B18] Woods D. Vangaveti S. Egbanum I. Sweeney A. M. Li Z. Bacot-Davis V. (2020). Conditional DnaB protein splicing is reversibly inhibited by zinc in mycobacteria. mBio 11. 10.1128/mBio.01403-20 PMC7360933 32665276

