# Supplementary material for: Controllable intein splicing and N-terminal cleavage at mesophilic temperatures
Source: Front Bioeng Biotechnol. 2025 Feb 7;13:1543573. doi: 10.3389/fbioe.2025.1543573 (PMC11842431; doi:10.3389/fbioe.2025.1543573)
Supplement: Supplementary file 1 [file DataSheet1.docx]

*Supplementary Material*

Controllable intein splicing and N-terminal cleavage at mesophilic temperatures

Taylor A. McNeal^1^, Joel Weinberger II^1^, Geraldy L. S. Liman^2^, Tia M. Ariagno^1^, David W. Wood^3^, Thomas J. Santangelo^2^, and Christopher W. Lennon^1*^

^1^Department of Biological Sciences, Murray State University, Murray, KY, United States

^2^Department of Biochemistry and Molecular Biology, Colorado State University, Fort Collins, CO, United States

^3^William G. Lowrie Department of Chemical and Biomolecular Engineering, The Ohio State University, Columbus, OH, United States

*** Correspondence:**Corresponding Author
clennon1@murraystate.edu


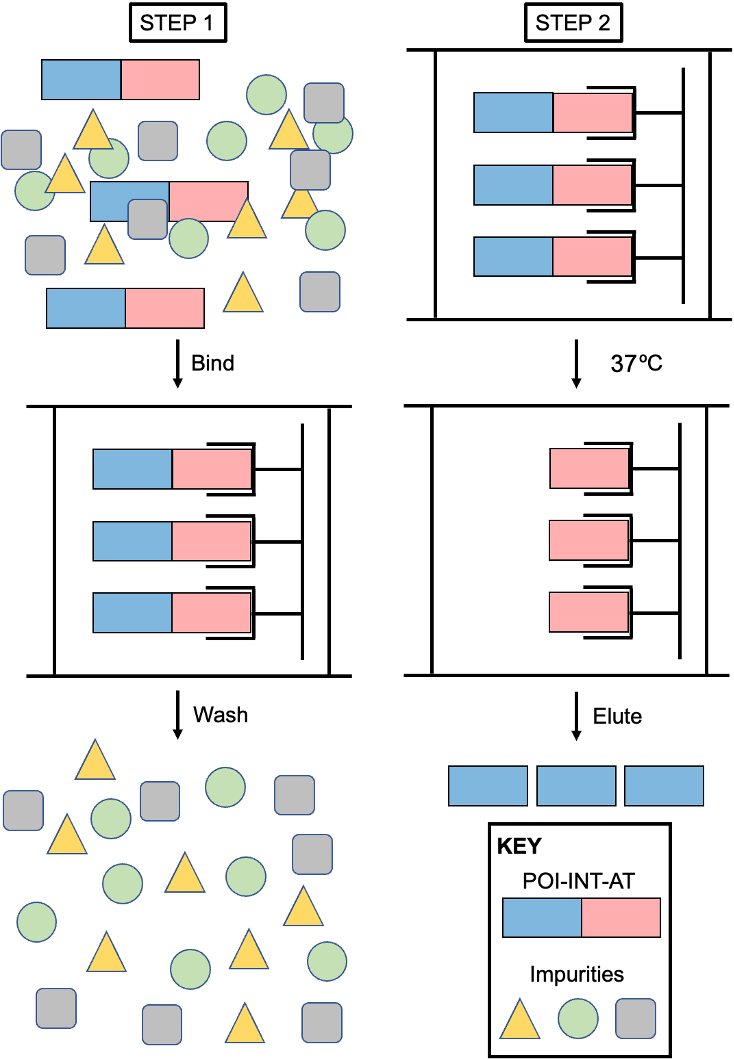


**Supplemental Figure 1: Cartoon of intein-based N-terminal cleavage protein purification strategy.** The protein of interest (POI) to be purified is shown in blue as the N-extein. The intein (INT) and affinity tag (AT) are shown in pink. In step 1, the POI-INT-AT is isolated from impurities using chromatography. In step 2, the POI is released from the INT-AT in a purified form by incubation at 37°C.

**
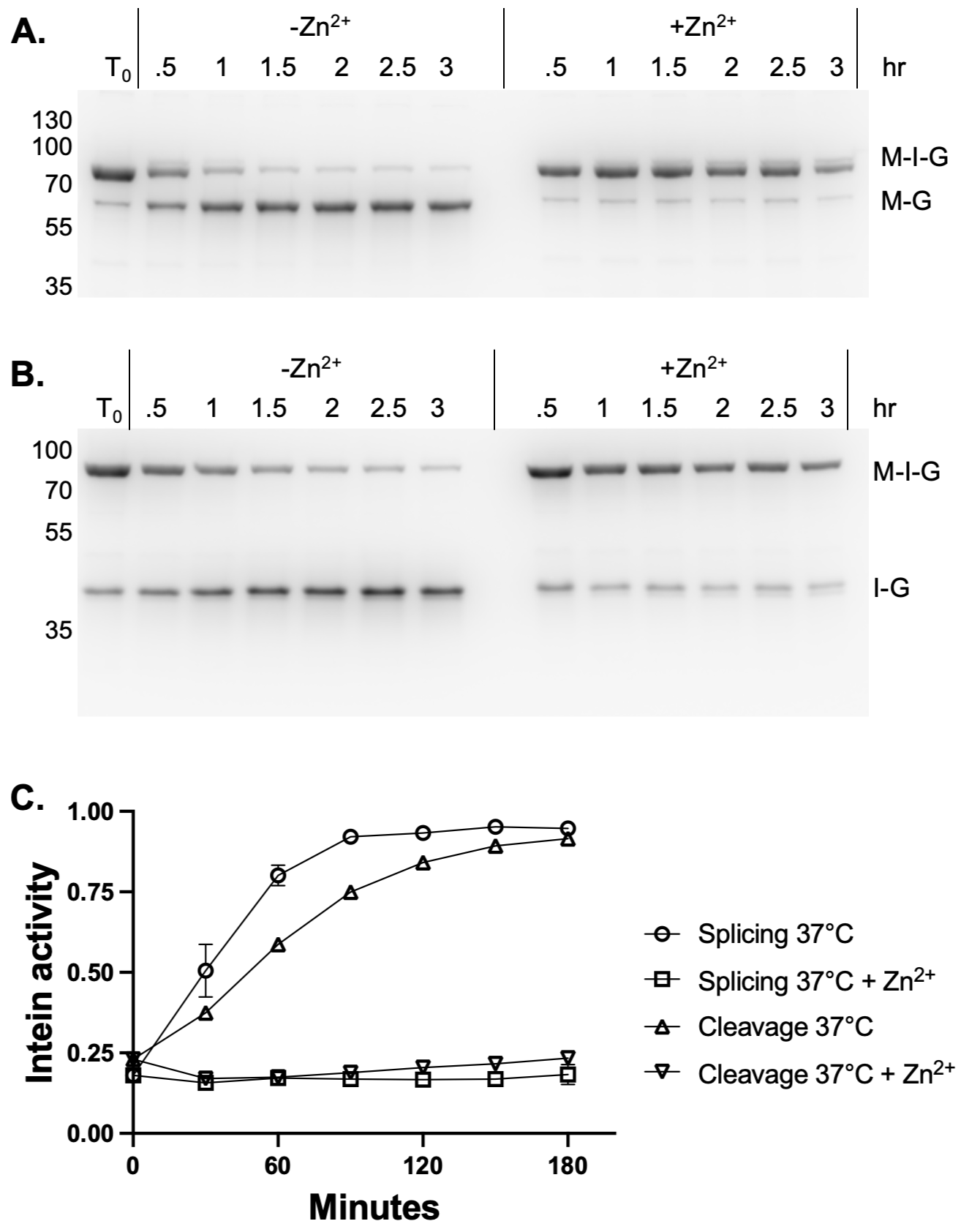
**

**Supplemental Figure 2: Zinc blocks TkDE splicing and TkDE-AA N-terminal cleavage (NTC). (A)** Splicing of TkDE and **(B)** NTC of TkDE-AA are blocked by 10 mM zinc. In panels A and B, samples were incubated at 37ºC for the indicated times, and precursor (M-I-G), ligated exteins (M-G), and intein-GFP (I-G) bands are labeled. **(C)** Quantification of TkDE splicing and TkDE-AA NTC in the presence or absence of zinc in panels A and B as described in Figure 1. When error bars are not shown in panel C, they are smaller than the symbol. Protein size markers are in kilodaltons.

**
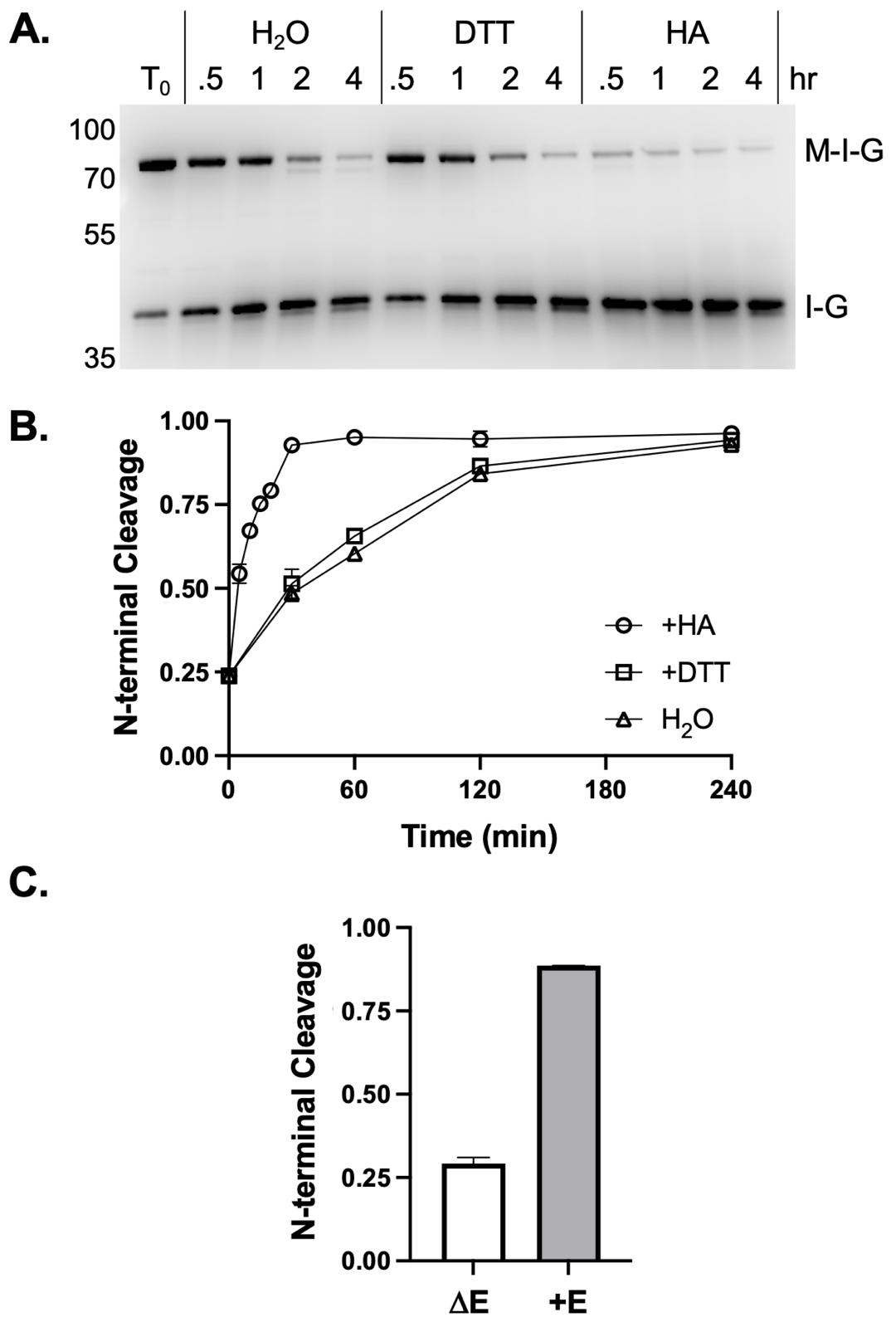
**

**Supplemental Figure 3: Hydroxylamine (HA), but not dithiothreitol (DTT), accelerates TkDE-AA NTC (NTC), and the homing endonuclease does not prevent NTC. (A)** TkDE-AA NTC at 37ºC in the absence of an external nucleophile (water) or presence of 50 mM DTT or HA. Precursor (M-I-G) and intein-GFP (I-G) bands are indicated. **(B)** Quantification of TkDE-AA NTC in the presence of water, DTT, or HA at indicated times as described in Figure 1. **(C)** Quantification of TkDE-AA and TkE-AA NTC following expression in *E. coli* at 15ºC for ~20 hours. When error bars are not shown in panels B and C, they are smaller than the symbol or line. Protein size markers are in kilodaltons.

**
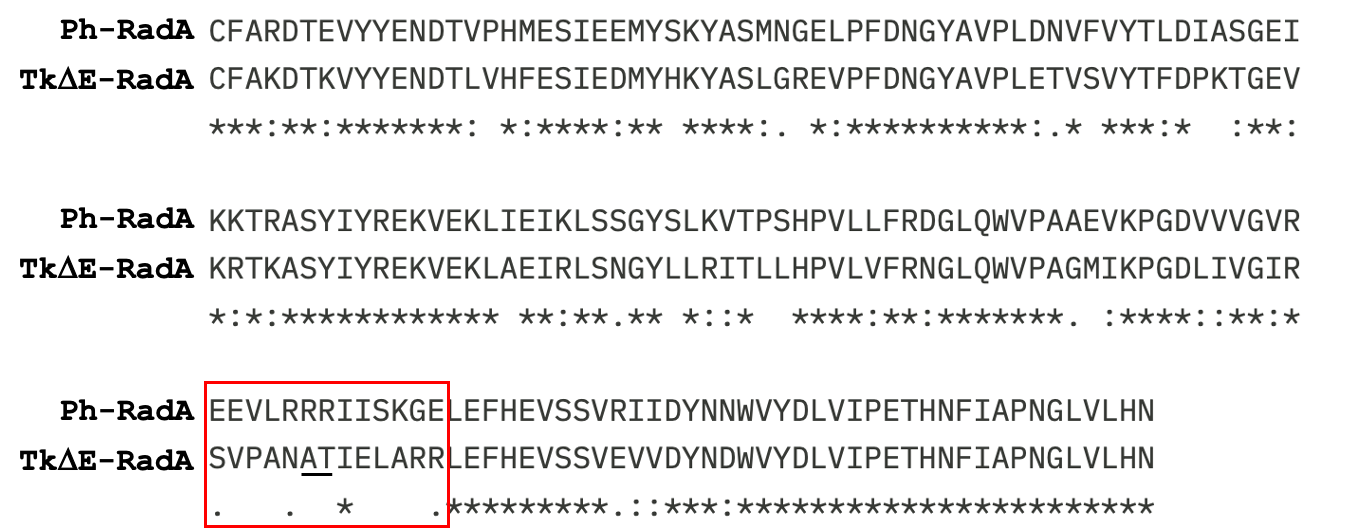
**

**Supplemental Figure 4: Clustal alignment of the TkΔE and Ph and RadA inteins.** The red box indicates the residues from the Ph intein exchanged with the TkΔE intein to form the TkPl intein. The site of homing endonuclease deletion within the TkDE intein is underlined. A “*” symbol indicates same reside, a “:” symbol indicates highly conserved residue, and a “.” symbol indicates a partially conserved residue.
